# Long noncoding RNA MALAT1 as a candidate serological biomarker for the diagnosis of non‐small cell lung cancer: A meta‐analysis

**DOI:** 10.1111/1759-7714.13265

**Published:** 2019-12-17

**Authors:** Jie Pan, Yuan Bian, Zhuo Cao, Limei Lei, Jiongwei Pan, Jinwei Huang, Xiaoping Cai, Xiang Lan, Hao Zheng

**Affiliations:** ^1^ Department of General Practice Medicine Lishui People's Hospital, the Six Affiliated Hospital of Wenzhou Medical University Lishui Zhejiang Province China; ^2^ Department of Respiratory Zhuji Affiliated Hospital of Shaoxing University Zhuji China; ^3^ Department of Respiratory Lishui People's Hospital, the Six Affiliated Hospital of Wenzhou Medical University Lishui Zhejiang Province China; ^4^ Department of Radiation Lishui People's Hospital, the Six Affiliated Hospital of Wenzhou Medical University Lishui Zhejiang Province China

**Keywords:** Long noncoding RNA, MALAT1, meta‐analysis, non‐small cell lung cancer: diagnosis, serological biomarker

## Abstract

**Background:**

To investigate the diagnostic efficacy of long noncoding RNA metastasis‐associated in lung adenocarcinoma transcript l (MALAT1) as a candidate serological biomarker for non‐small cell lung cancer (NSCLC).

**Methods:**

Diagnostic studies relevant to circulation long noncoding RNA MALAT1 as a candidate serological biomarker for NSCLC were electronically systematically searched in PubMed, EMBASE, EBSCO and CNKI databases. Suitable studies were included in the meta‐analysis by pooling the diagnostic sensitivity, specificity, positive likelihood ratio (+LR), negative likelihood ratio (−LR), diagnostic odds ratio (DOR) and area under the symmetric ROC curve (AUC) through a random or fixed effects model. Deeks' funnel plot was applied for publication bias evaluation.

**Results:**

Six studies with eight datasets were finally included in the meta‐analysis after a systematic search of the databases was performed. The pooled diagnostic sensitivity, specificity, +LR, −LR and DOR were 0.81 (95% CI:0.78–0.84), 0.67 (95% CI:0.63–0.71), 2.61 (95% CI:1.81–3.71), 0.28 (95% CI:0.19–0.43) and 13.73 (95% CI:6.19–30.44), respectively. The pooled area under the ROC curve (AUC) were 0.8663 and 0.8658, respectively by symmetric and asymmetric methods.

**Conclusion:**

Based on the results of our study, serum long noncoding RNA MALAT1 is a promising biomarker for NSCLC screening. However, due to its low specificity, MALAT1 positive cases need further validation for NSCLC by other diagnostic methods such as radiology, cytology, etc.

## Introduction

Carcinoma of the lung is the most diagnosed malignant tumor in men and second in women and the leading cause of cancer‐related deaths in both males and females.^1^, ^2^ According to the pathology type, lung cancer is generally divided into small cell lung cancer (SCLC) and non‐small cell lung cancer (NSCLC) which is further divided into squamous cell lung carcinoma and adenoma lung carcinoma. The general prognosis of NSCLC is poor due to the advanced stage at which it is first diagnosed in 80% of all NSCLC cases. Due to its advanced clinical stage, most patients do not have the option of surgery and chemoradiotherapy is the treatment of choice.^3^ Therefore, early diagnosis is important for the prognosis of patients diagnosed with NSCLC. However, early diagnosis or screening for lung cancer is difficult due to the lack of effective methods.^4^, ^5^, ^6^

Because of minimal invasion and easy sample collection, serological biomarkers for lung cancer diagnosis or screening are of great interest.^7^, ^8^, ^9^, ^10^ Several studies have recently evaluated long noncoding RNA MALAT1 expression in serum or plasma of NSCLC patients and discussed its diagnostic performance.^11^, ^12^ However, due to limited statistical power caused by a small sample size, the findings of the publications were inconclusive. Therefore, we performed a meta‐analysis by pooling the open published studies relevant long noncoding RNA MALAT1 as a candidate serological biomarker for NSCLC.

## Methods

### Publication search

Diagnostic clinical studies on long noncoding RNA MALAT1 as a candidate serological biomarker for the diagnosis of NSCLC were systematically searched in the electronic databases of PubMed, EMBASE, EBSCO and CNKI. The electronic search words used were as follows: “lung carcinoma/lung cancer/lung tumor/lung neoplasm/malignant neoplasm of lung/non‐small cell lung cancer/NSCLC” and “metastasis associated in lung adenocarcinoma transcript 1/MALAT1/MALAT‐1/lncRNA MALAT 1/lncRNA MALAT‐1.” The reference to include published data was also screened in order to find potential suitable studies.

### Inclusion and exclusion criteria

The publication inclusion criteria was defined as: (i) The patients included in each original study were confirmed with non‐small cell lung cancer by cytology or pathology; (ii) the controls were healthy subjects or cases without malignant disease; (iii) MALAT1 in serum or plasma was examined by real‐time PCR assay; (iv) the number of true positive (tp), false positive (fp), false negative (fn) and true negative (tn) in NSCLC and controls could be drawn or calculated from the original included studies. Exclusion criteria included: (i) small cell lung cancer or other type of lung carcinoma; (ii) not enough data to draw the tp, fp, fn and tn and (iii) MALAT1 detected in tissue or bronchoalveolar lavage fluid other than serum or plasma.

### Data collection

The data and main information from each included study was extracted by two reviewers (Jie P and Yuan B) independently and cross‐checked. The extracted information and data included the first and corresponding author, period of the work performed, area of the study performed, number of tp fp fn tn cases, control type and the cutoff value of serum MALAT1 level.

### Statistical analysis

Statistical analysis was performed using MetaDiSc1.4 (http://www.biomedsearch.com) statistical software. χ^2^ and I^2^ test were applied in order to evaluate the statistical heterogeneity across the included publications. The diagnostic effect size (sensitivity, specificity, +LR, −LR, and DOR) were pooled through a random or fixed effects model according to the statistical heterogeneity evaluation. Deeks' funnel plot was applied for publication bias evaluation.

## Results

### General features of studies

**Figure 1:**
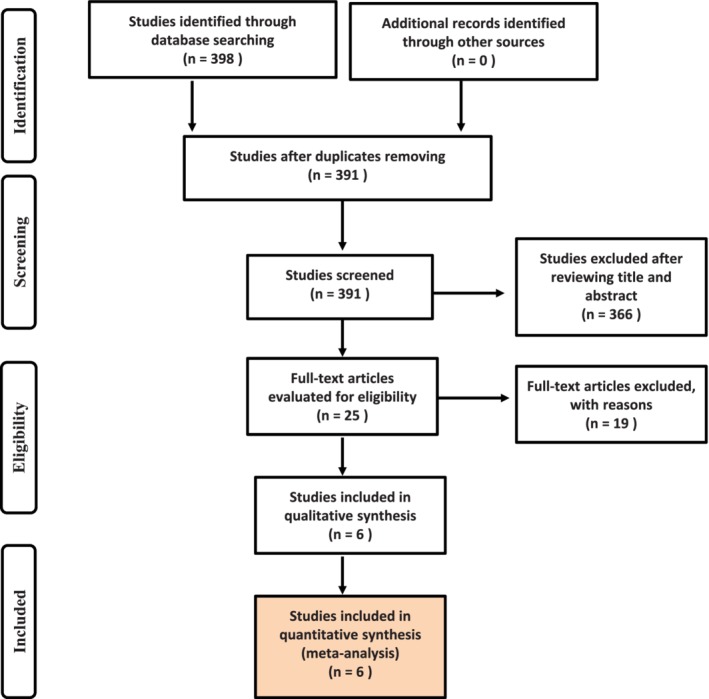
Flowchart of relevant studies from the electronic databases.

Six studies^11^, ^12^, ^13^, ^14^, ^15^, ^16^ with eight datasets were finally included in the meta‐analyses after a systematic search of the databases was performed. The identification process of the studies is shown in Figure 1. Of the six diagnostic clinical studies included, two used non‐cancer subjects as the control and four used healthy subjects as the control. All NSCLC patients had been diagnosed by pathology or cytology. The main features of the included six studies are shown in Table 1.

**Table 1:** General characteristics of the 19 publications included in the study

		Sample size		Distribution					
First author	Year	NSCLC	Control	TP	FP	FN	TN	Control type	Cutoff value
Shi	2016	60	92	53	5	8	87	Noncancer control	0.62
Guo	2015	88	100	72	22	29	43	Healthy control	10.34
Weber	2013	45	25	21	0	24	25	Noncancer control	Maximum Youden's Index:‐0.41
Wen	2016	60	60	50	21	10	99	Healthy control	0.03
Peng	2016	36	36	32	17	4	19	Healthy control	1.10
Peng	2016	120	71	119	46	1	25	Healthy control	2.08
Guo	2019	148	117	118	56	30	61	Healthy control	Maximum Youden's Index:0.51
Guo	2019	120	103	95	41	25	62	Healthy control	Maximum Youden's Index:0.51

NSCLC, non‐small cell lung cancer; TP, true positive; FP, false positive; FN, false negative; TN, true negative

### Pooled results

**Figure 2:**
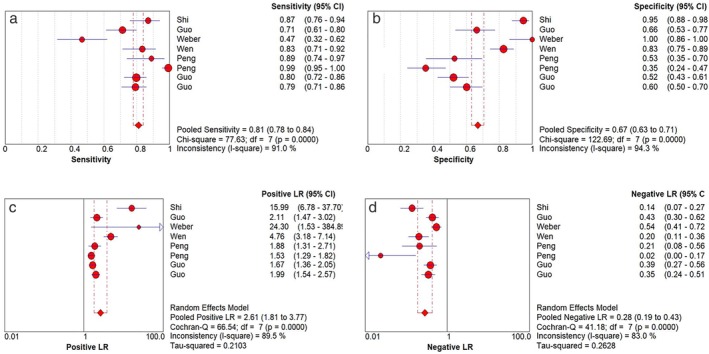
Forest plot of diagnostic effective size for MALAT1 as a candidate serological biomarker for non‐small cell lung cancer (NSCLC). (**a**) pooled diagnostic sensitivity; (**b**) pooled diagnostic specificity; (**c**) pooled +LR; (**d**) pooled −LR.

**Figure 3:**
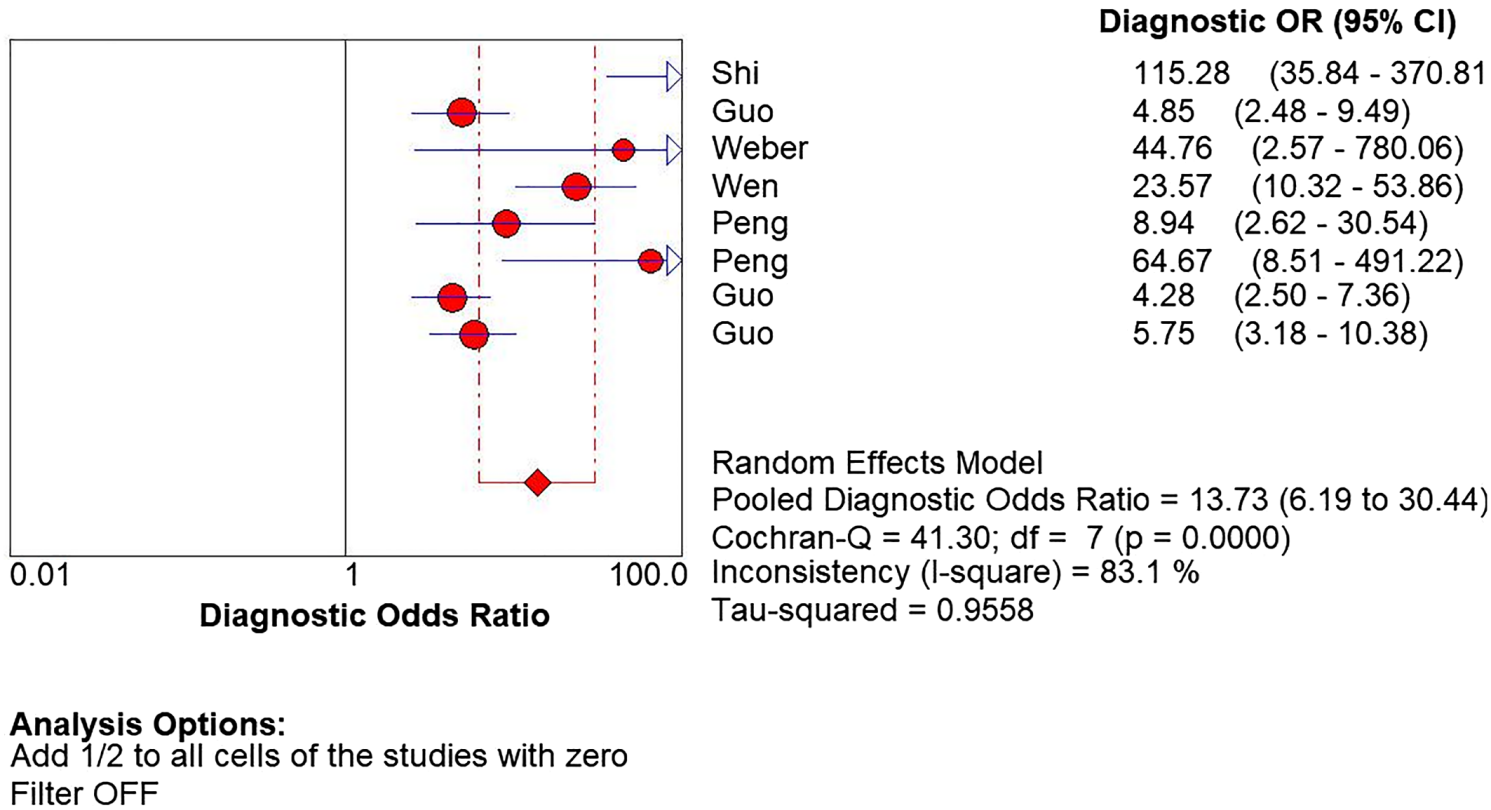
Forest plot of diagnostic odds ratio for MALAT1 as a candidate serological biomarker for non‐small cell lung cancer (NSCLC).

Before pooling the diagnostic effect size of sensitivity, specificity, +LR and −LR, the statistical heterogeneity across the included studies was evaluated. The results indicated significant statistical heterogeneity existed for all the diagnostic effect size (*P* < 0.05). Therefore, the data was pooled using a random effects model. The pooled diagnostic sensitivity, specificity, +LR and −LR were 0.81(95% CI:0.78–0.84), 0.67(95% CI:0.63–0.71), 2.61(95% CI:1.81–3.71) and 0.28 (95% CI:0.19–0.43), respectively, Figure 2. The pooled DOR was 13.73 (95% CI:6.19–30.44) using a random effects model, Figure 3.

### Pooled ROC

**Figure 4:**
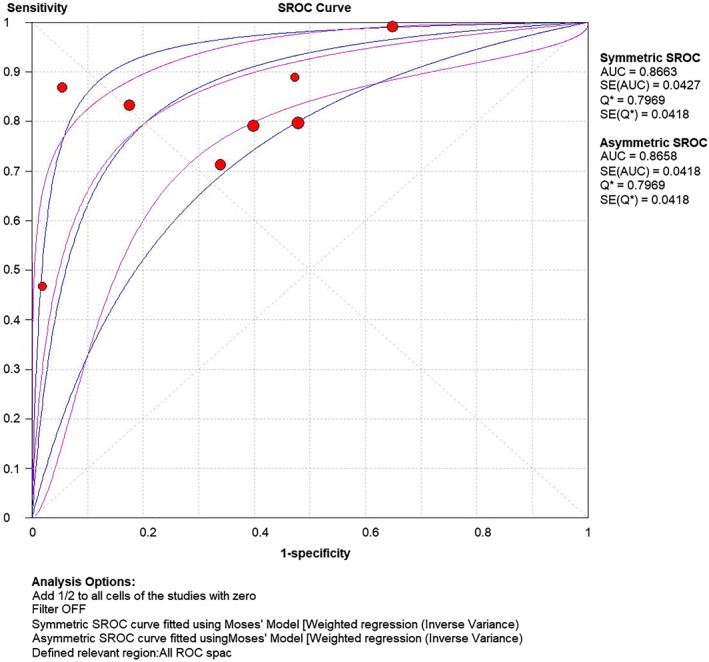
ROC curve of MALAT1 in serum as a biomarker for the diagnosis of non‐small cell lung cancer (NSCLC).

The area under the ROC curve (AUC) was pooled by symmetric or asymmetric methods with results of AUC = 0.8663 and 0.8658, respectively, Figure 4.

### Subgroup analysis

According to the pathology type of NSCLC, subgroup analysis for squamous cell lung cancer and lung adenocarcinoma was made. The pooled sensitivity, specificity, +LR, −LR and DOR for squamous cell lung cancer and lung adenocarcinoma are shown in Table 2.

**Table 2:** Subgroup analysis of MALAT1 in serum as a biomarker for non‐small cell lung cancer (NSCLC) diagnosis according to pathology type

Diagnostic reference	Squamous cell carcinoma	Chi‐square/Cochran‐Q	*P*‐value	Adenocarcinoma	Chi‐square	*P*‐value
Sen	0.74 (0.60–0.85)	10.10	0.0015	0.62 (0.48–0.75)	8.13	0.0043
Sep	0.87 (0.78–0.93)	8.33	0.0039	0.88 (0.79–0.94)	7.51	0.0061
+LR	8.01 (1.36–47.31)	2.01	0.156	5.44 (2.01–14.70)	1.19	0.2760
−LR	0.23 (0.03–1.84)	9.10	0.0027	0.43 (0.17–1.10)	7.10	0.0077
DOR	54.86 (13.65–220.43)	0.00	0.9453	17.55 (6.57–46.89)	0.20	0.6583

### Publication bias evaluation

**Figure 5:**
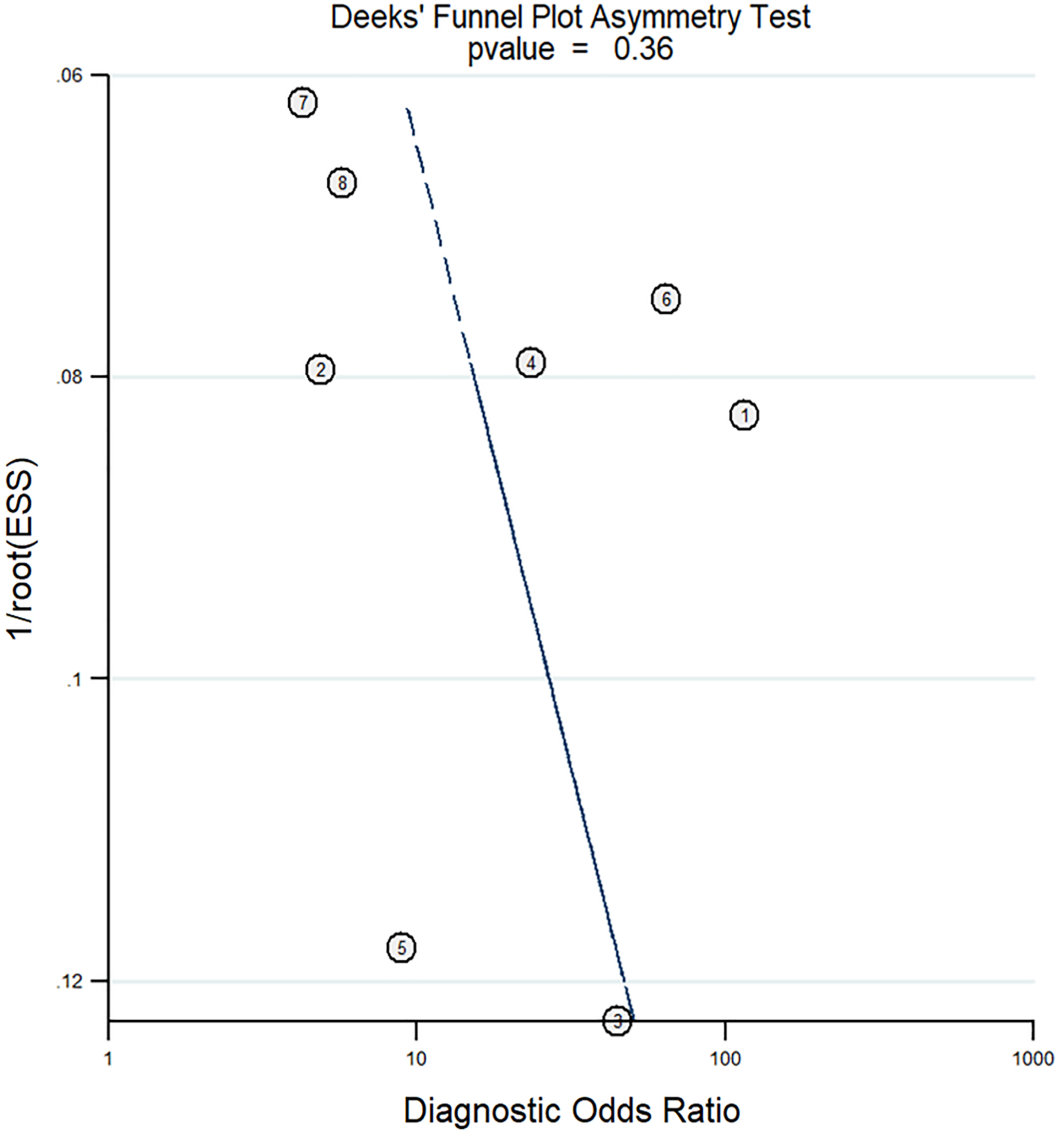
Deeks' funnel plot indicated no publication bias. (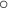) Study and (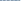) regression line.

The publication bias was evaluated by Deeks' funnel plot and indicated no significant publication bias for MALAT1 as a candidate serological biomarker for NSCLC (*P* > 0.05), Figure 5.

## Discussion

Long noncoding RNA belongs to one of the main types of noncoding RNA family. In recent years, many studies have found that abnormal expression of long noncoding RNA MALAT1 can be used as a potential biomarker of cancer diagnosis.^12^, ^13^ MALAT1 is one of the first identified long noncoding RNA related to post‐transcription modification. In 2003, Ji *et al*. found that the expression level of MALAT1 in metastatic tissue of NSCLC patients was significantly higher than that in nonmetastatic cancer tissue.^17^ The findings of the study by Zhao *et al*. indicated that MALAT1 may be an important molecule associated with lung cancer metastasis.^18^ At present, there are several meta‐analysis relevant to MALAT1 expression and patients’ prognosis with positive results.^19^, ^20^ However, there is no confirmed evidence of MALAT1 expression and cancer diagnosis. In the present meta‐analysis, the results showed that the sensitivity and specificity of serum MALAT1 in the diagnosis of lung cancer were 0.81 and 0.67, respectively, and the AUC was 0.6, indicating that the detection of MALAT1 in circulating blood was of high diagnostic value for lung cancer. Due to the high diagnostic sensitivity of serum MALAT1 as a reference for NSCLC, the misdiagnosis rate of lung cancer is low, which indicates that serum MALAT1 has better clinical application value in lung cancer screening. In addition, the diagnostic odds ratio (DOR) is also an important index to evaluate diagnostic efficiency. When the value is more than one, the larger the value is, the better the discrimination effect of the diagnostic test is.^12^ In this study, the overall combined DOR value was 13.73, suggesting that the detection of serological MALAT1 was more effective in the overall diagnosis of lung cancer. Furthermore, the combined positive likelihood ratio suggested that the probability of positive expression of MALAT1 in lung cancer patients was 2.6 times higher than that in the control group, which further reflected the potential value of circulating MALAT1 in the diagnosis of lung cancer.

The meta‐analysis in our study also had its limitations. First, only six studies with eight datasets were included in the present work. Second, the diagnostic sensitivity, specificity, +LR, −LR and DOR were pooled through a random effects model due to significant statistical heterogeneity. Third, only studies published in English or Chinese were screened and included in the meta‐analysis.

## Disclosure

No authors report any conflict of interest.
